# Comparison of hypoxia- and hyperoxia-induced alteration of epigene expression pattern in lungs of *Pleurodeles waltl* and *Mus musculus*

**DOI:** 10.1371/journal.pone.0299661

**Published:** 2024-02-28

**Authors:** Md. Mahmudul Hasan, Reiko Sekiya, Xu Zhang, Mhd Yousuf Yassouf, Tao-Sheng Li

**Affiliations:** 1 Department of Stem Cell Biology, Nagasaki University Graduate School of Biomedical Sciences, Sakamoto, Nagasaki, Japan; 2 Department of Stem Cell Biology, Atomic Bomb Diseases Institute, Nagasaki University, Sakamoto, Nagasaki, Japan; Laboratoire de Biologie du Développement de Villefranche-sur-Mer, FRANCE

## Abstract

Epigenetics is an emerging field of research because of its involvement in susceptibility to diseases and aging. Hypoxia and hyperoxia are known to be involved widely in various pathophysiologies. Here, we compared the differential epigene expression pattern between *Pleurodeles waltl* and *Mus musculus* (commonly known as Iberian ribbed newt and mouse, respectively) exposed to hypoxia and hyperoxia. Adult healthy newts and mice were exposed to normobaric hypoxia (8% O_2_) and hyperoxia (80% O_2_) for 2 hours. We collected the lungs and analyzed the expression of hypoxia-inducible factor 1 alpha (*Hif1α*) and several key epigenes from DNA methyltransferase (DNMT) family, histone deacetylase (HDAC) family, and methyl-CpG binding domain (MBD) family. The exposure to hypoxia significantly increased the mRNA levels of DNA methyltransferase 3 alpha (*Dnmt3α*), methyl-CpG binding domain protein 2 (*Mbd2*), *Mbd3*, and histone deacetylase 2 (*Hdac2*) in lungs of newts, but decreased the mRNA levels of DNA methyltransferase 1 (*Dnmt1*) and *Dnmt3α* in lungs of mice. The exposure to hyperoxia did not significantly change the expression of any gene in either newts or mice. The differential epigene expression pattern in response to hypoxia between newts and mice may provide novel insights into the prevention and treatment of disorders developed due to hypoxia exposure.

## Introduction

Epigenetics represents the machinery that regulates transcription of genes and is the result of addition or deletion of epigenetic modifications [1]. The most basic epigenetic modifications are methylation to DNA and core histone proteins. Beside methylation (addition of methyl groups to the arginine or lysine residues), the histone proteins are also vulnerable to different modifications including acetylation (incorporation of an acetyl group to lysine molecules residing in the protruding tails), phosphorylation (addition of phosphate group to serine, threonine, and tyrosine amino acid usually in the N-terminal tails), SUMOylation (covalently linking a small ubiquitin-like modifier SUMO to a specific lysine amino acid), and ubiquitination (transportation of ubiquitin protein to the core histone). The aforementioned modifications regulate chromatin accessibility to transcription factors and enzymes that are responsible for transcription initiation [1]. Epigenetics is known to play key roles in the development of organisms and diseases [2]. Environmental cues including temperature, oxygen level, chemical pollutants, etc. have impact on epigenetics and disease susceptibility [3–5].

Hypoxia, the supply of lower amount of oxygen compared to required level, is detrimental for most of the vertebrates, as it dramatically impairs cellular metabolic demand. Almost all animals have the chance of being exposed to environmental hypoxia as it is a widely existing environmental condition. On the other hand, hyperoxia is the supply of elevated level of oxygen compared to the normal level. Supply of excess oxygen is common for patients undergoing surgery after general anesthesia and those who are suffering from acute or life-threatening illness. Both of the hypoxia and hyperoxia induce severe oxidative stress in the lung [6–8]. Hypoxia induces pulmonary hypertension, interstitial pulmonary fibrosis, acute respiratory distress syndrome, and chronic obstructive pulmonary disease [7,9]. Elevated oxygen supply to preterm infant induces bronchopulmonary dysplasia (BPD) [10]. Hyperoxia-induced BPD has been reported due to acetylation of histone H3 lysine 27 (H3K27) and the subsequent transcription activation of *Cdkn1α* [11]. Effective and rapid therapeutic intervention is an urgent need to get rid of the pathophysiological complication induced by hypoxia and hyperoxia. As epigenetic modifications are reversible, targeting epigenetic landmarks involved in a disease process may offer novel approach to planning effective and rapid therapies. For example, intermittent hypoxia-induced hypermethylation of CpG near the transcription start site of superoxide dismutase 2 (*Sod2*) gene suppresses its expression in mice and mitigation of this transcription inhibition by treating with DNA methyltransferase (DNMT) inhibitor decitabine has been reported [12]. Therefore, studying the epigenetics of hypoxia and hyperoxia tolerant animals may offer clues to develop preventive measures or treatment options to get rid of hypoxia- and hyperoxia-induced pathological conditions in human.

Compared to mammals, the ectothermic vertebrates (e.g. amphibians, fishes, and reptiles) are more tolerant to hypoxia and hyperoxia [13–15]. Some fishes (*Astronotus ocellatus*, *Carassius carassius* and *Hemiscyllium ocellatum*) and turtles (*Chrysemys picta* and *Trachemys scripta elegans*) can survive for several hours to days without any amount of oxygen [15–18]. For instance, adult *A*. *ocellatus* can survive approximately 6 hours without oxygen [16], while *Chrysemys* turtle can tolerate anoxia for more than 60 hours [17].

*Pleurodeles waltl*, member of *Salamandridae* family, is an ectothermic animal with excellent organ regeneration capacity. *P*. *waltl* is generally called as newt. The regeneration capacity of newts is age independent [19]. Another interesting property of newts is resistance to carcinogenesis [20,21]. As they are semiaquatic animals, their life cycle follows three developmental stages, such as larvae (aquatic) stage, juvenile (terrestrial) stage, and adult (semiaquatic) stage [22]. Similar to having different developmental phases and habitats, they have different ways of breathing. At larvae stage, they use their feathery gills to breathe under water. Following metamorphosis, the larvae turn into juvenile stage and develop legs and lungs for surviving on land. This respiratory transition process is unique to amphibians [23]. They live on land from months to years to become adult from juvenile phase. After being adult, they can live both on land and in water, representing them as semiaquatic. Except the lungless newts, the adult one uses lungs for breathing on surface and commonly they follow cutaneous respiration in water.

In a previous article by our group, increased acetylation to histone H3 lysine 9 (H3K9), H3K14, and H3K27 has been reported in the regenerating tail tissue of *P*. *waltl* [24]. It has been reported that semiaquatic freshwater turtle *T*. *scripta* elegans develops tolerance to oxygen starvation by harboring DNA hypermethylation and subsequent global transcription inhibition [18]. Thus, we hypothesized that the epigenetics in salamander is different than in mammal and we designed this study to compare the differential epigene expression pattern in lungs of newts and mice in response to hypoxia and hyperoxia exposures.

## Materials and methods

### Animal care

Iberian ribbed newts (*P*. *waltl*) and C57BL/6 mice (CLEA, Japan) were used in this study. Newts were obtained from the Tottori University [25] and the Hiroshima University Amphibian Research Center through a National BioResource Project (NBRP) of the Ministry of Education, Culture, Sports, Science and Technology (MEXT), Japan. Nine male adult newts (4 to 5 years old) and nine male adult mice (12 to 14 weeks old) were used for this experiment. Newts were kept in a temperature controlled room (25 ± 1.5°C) with 12:12-h light–dark cycle. They were housed in polypropylene cage filled with tap-water and equipped with continuous air supply into the water. Water was changed three times in a week after feeding them catfish pellet food (Kyorin Food Industry Co., Ltd., Japan). Mice were housed in a pathogen-free and temperature controlled room with 12:12-h light–dark cycle. Mice were accessed to food and water *ad libitum*. Approval of this study was taken from Institutional Animal Care and Use Committee, Nagasaki University (memo no. 2017–1). All animal procedures were executed according to the institutional and national guidelines.

### Exposing animals to hypoxia and hyperoxia

Newts and mice were exposed to modified oxygen in air. The incubators were maintained at 25 ± 0.5°C temperature with 5% CO_2_ supply. Animals were exposed to 20% O_2_ (normoxia), 8% O_2_ (hypoxia), and 80% O_2_ (hyperoxia) for 2 hours. Exposing animals to modified oxygen was performed individually following three replications. Then, they were sacrificed following cervical dislocation and their lungs were collected and stored immediately at -80°C.

### Reverse transcription-quantitative polymerase chain reaction

RNA was isolated from lung tissue using Direct-zol™ RNA MiniPrep Kits (Zymo Research, USA) following steps described in manufacturer’s protocol. Quality and quantity of RNA was evaluated using a NanoDrop™ 2000/2000c spectrophotometer (Thermo Scientific). The isolated RNA (2 μg) was reverse-transcribed using SuperScript™ VILO™ MasterMix (Invotrogen, USA) in 20 μL reaction mixture. Quantitative polymerase chain reaction (qPCR) was performed using THUNDERBIRD™ SYBR^®^ qPCR Mix (TOYOBO, Japan) on a Bio-Rad CFX96 real-time PCR detection system (Bio-Rad, USA). Complementary DNA (cDNA) equivalent to 50 ng RNA (except 12.5 ng for *Hif1α* and corresponding *Gapdh* in case of mice) was used in each 20 μL PCR reaction. Efficiency of PCR was confirmed based on parallelism of the geometric slops on the amplification plot. The melt curve analysis was performed according to the set program on Bio-Rad CFX96 real-time PCR detection system (Bio-Rad, USA). Primers (Table 1) used in this study were synthesized by Hokkaido System Science Co., Ltd. (Japan). Of note, the primers for newt were designed based on the transcript database iNewt (http://www.nibb.ac.jp/imori/main/) developed by Matsunami et al. [26]. We used the basic local alignment search tool (BLAST) of that database using the corresponding transcript FASTA sequence of mouse against the transcript dataset “Trinity_Pwal_v2.fasta” to find out the target transcript. Thereafter, the best matched FASTA sequences were downloaded and used to design primers utilizing the National Center for Biotechnology Information (NCBI) primer designing tool. However, normalized fold change calculation was performed using endogenous glyceraldehyde 3-phosphate dehydrogenase (*Gapdh*) gene based on ΔΔCq method [27].

**Table 1:** List of primers.

Genes	Primers	Sequences (5’-3’)	
		Newt	Mouse
*Hif1α*	Forward	GGTGAAGACCGAGCCAAGAA	ACCTTCATCGGAAACTCCAAAG
	Reverse	CGAACTGTCGCTGGTGTTTG	CTGTTAGGCTGGGAAAAGTTAGG
*Dnmt1*	Forward	TGCTTACTGCGACCACTACC	CCGTGGCTACGAGGAGAAC
	Reverse	AGAGAACGCTACCAAACGCA	TTGGGTTTCCGTTTAGTGGGG
*Dnmt3α*	Forward	AACCCTACGTTCACGCAAGT	GGCCGAATTGTGTCTTGGTG
	Reverse	TTATTGGGGACTGGGCTAGG	CCATCTCCGAACCACATGAC
*Dnmt3β*	Forward	GCTGGATTGGGCATTTGGAG	AGCGGGTATGAGGAGTGCAT
	Reverse	GTCGTTTAGGGAGTGGGCAG	GGGAGCATCCTTCGTGTCTG
*Mbd1*	Forward	GCACTTCCTCAGGAGCCAAT	AAAGTTGAGCTGACTCGGTACT
	Reverse	AGAGCCCTACTGGGGAGAAG	TCTTGGCTGGTTTAGAAGGCT
*Mbd2*	Forward	ATCTCGACAACTGGGGCTTG	AGAACAAGGGTAAACCAGACCT
	Reverse	CGGCAAAAGCGATGTCTACT	ACTTCACCTTATTGCTCGGGT
*Mbd3*	Forward	TGGTATATGGCGAAGAATGTTGC	CCCCAGCGGGAAGAAGTTC
	Reverse	AGCCGTGTGCACTTCATTCA	CGGAAGTCGAAGGTGCTGAG
*Mecp2*	Forward	ACCAATCGTCAGGGGAGAGA	ATGGTAGCTGGGATGTTAGGG
	Reverse	AGTGTGCAGTTCCAAGGCTC	TGAGCTTTCTGATGTTTCTGCTT
*Hdac1*	Forward	TGGAACTTGGCCTGGATTAGG	TGAAGCCTCACCGAATCCG
	Reverse	TGCAGTTCAAGTCGTCTGGT	GGGCGAATAGAACGCAGGA
*Hdac2*	Forward	ACTGACCAACCCAGTAACCCA	GCTTGCCATCCTCGAATTACT
	Reverse	GGCCAGTTCCGCTCACTACA	GTCATCACGCGATCTGTTGTAT
*Gapdh*	Forward	CGGAATCAACGGATTTGG	TGGCCTTCCGTGTTCCTAC
	Reverse	GCGTCCATGGGTAGAGTCAT	GAGTTGCTGTTGAAGTCGCA
*α-actin*	Forward	TGGTCGTGACCTGACTGAT	-
	Reverse	TCACGGACAATCTCACGTTC	-
*β-actin*	Forward	AAGAAGGTTGGAAGAGCGCC	-
	Reverse	TCTGGACTTCGAGCAGGAGA	-
*Taf6*	Forward	TTCACGAGCTGTCTGTGGAG	-
	Reverse	CCTGGGAAGCATTTGGTAGA	-
*Ef1α*	Forward	AACATCGTGGTCATCGGCCAT	-
	Reverse	GGAGGTGCCAGTGATCATGTT	-
*mtRNA16s*	Forward	CGTGCAGAAGCGGAGATAA	-
	Reverse	TGTCGGGCTGTTGTAGGG	-

### Statistical analysis

The results in this study are presented as the mean ± SD (standard deviation). The one-way analysis of variance (ANOVA) with Dunnett’s multiple comparison test was performed on GraphPad Prism (version 8) software.

## Results

### Hypoxia does not affect expression of hypoxia-inducible factor 1 alpha (*Hif1α*) in newts and mice

**Fig 1:**
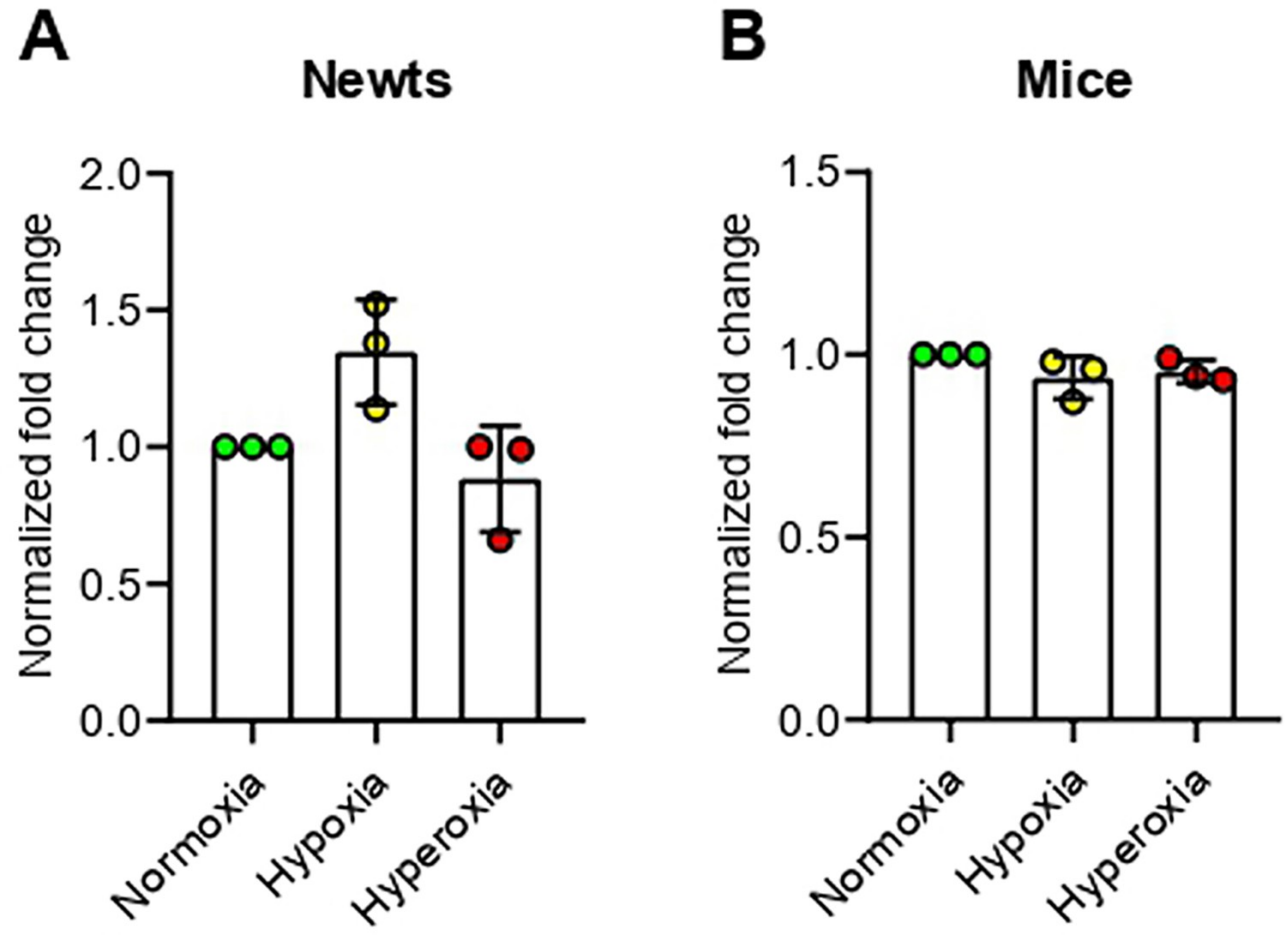
Effect of hypoxia and hyperoxia on expression of *Hif1α* gene in newts (A) and mice (B). Data are presented as mean ± SD. Experiments were done in triplicate.

Firstly, we checked expression of hypoxia-inducible factor 1 alpha (*Hif1α*) mRNA. Though the change of *Hif1α* mRNA expression was not significant compared to normoxia, we observed an increasing trend only in newts at hypoxia (Fig 1A). The level of *Hif1α* mRNA was not changed among groups in mice (Fig 1B).

### Hypoxia up-regulates DNA methyltransferases (DNMTs) in newts, but conversely down-regulates in mice

**Fig 2:**
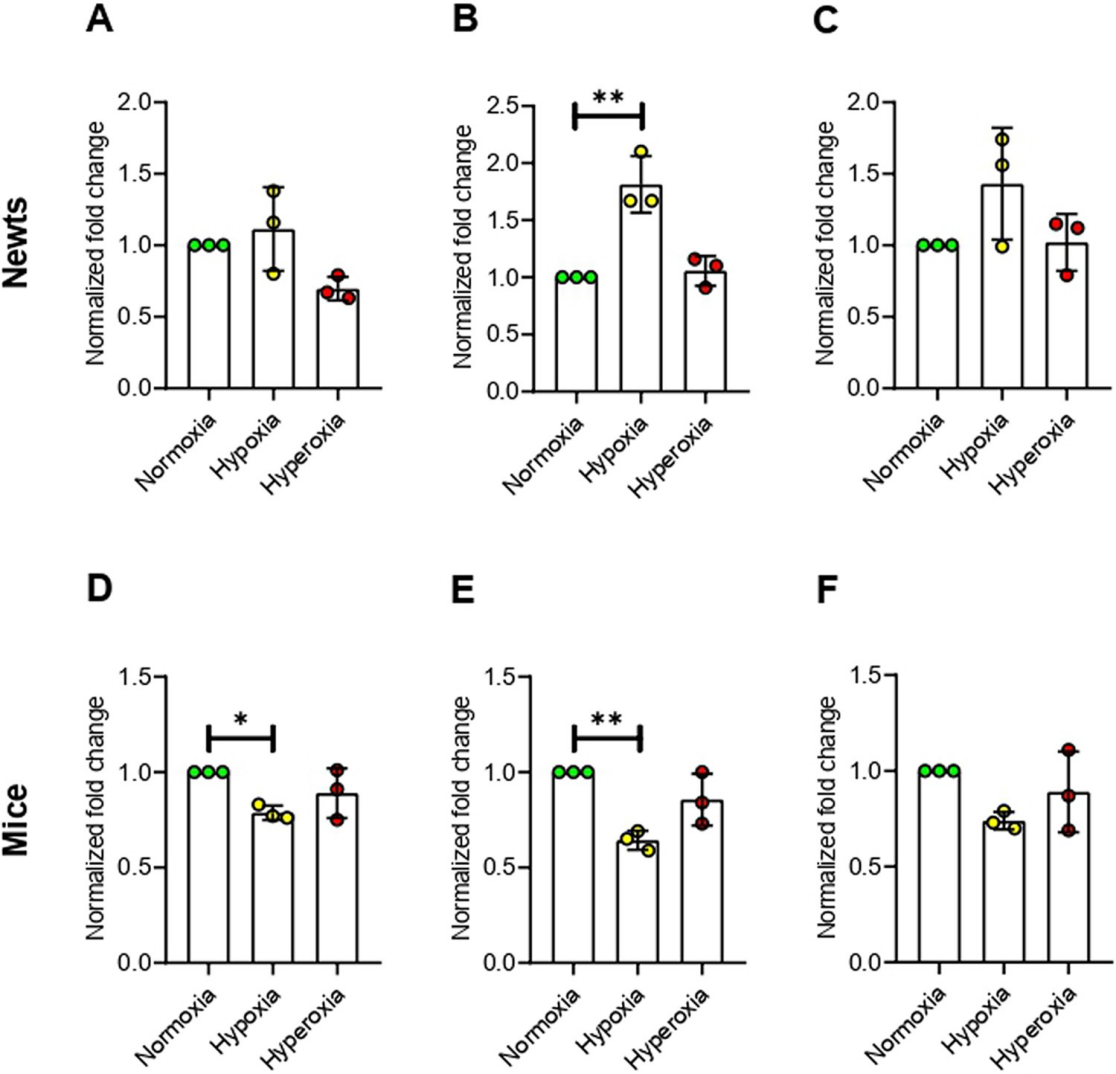
Effect of hypoxia and hyperoxia on genes that encode DNA methyltransferases (DNMTs) in newts (A, B, and C) and mice (D, E, and F). A and D = DNA methyltransferase 1 (*Dnmt1*), B and E = DNA methyltransferase 3 alpha (*Dnmt3α*), C and F = DNA methyltransferase 3 beta (*Dnmt3β*). Data are presented as mean ± SD. Experiments were done in triplicate. *p<0.05, **p<0.01.

According to our results, DNA methyltransferase 1 (*Dnmt1*) expression was not changed significantly at any condition of oxygen treatment in newts (Fig 2A). On the other hand, expression of *Dnmt1* was decreased significantly (*p*<0.05) in mice at hypoxia, but not changed at hyperoxia (Fig 2D). At hypoxic condition, expression of DNA methyltransferase 3 alpha (*Dnmt3α*) was increased significantly (*p*<0.01) in newts (Fig 2B), but decreased significantly (*p*<0.01) in mice (Fig 2E). *Dnmt3α* expression was not affected in any species due to hyperoxia exposure (Fig 2B and 2E). No significant change was observed in *Dnmt3β* expression in any animal exposed to hypoxia or hyperoxia (Fig 2C and 2F).

### Hypoxia up-regulates methyl-CpG binding domains (MBDs) in newts, but not in mice

**Fig 3:**
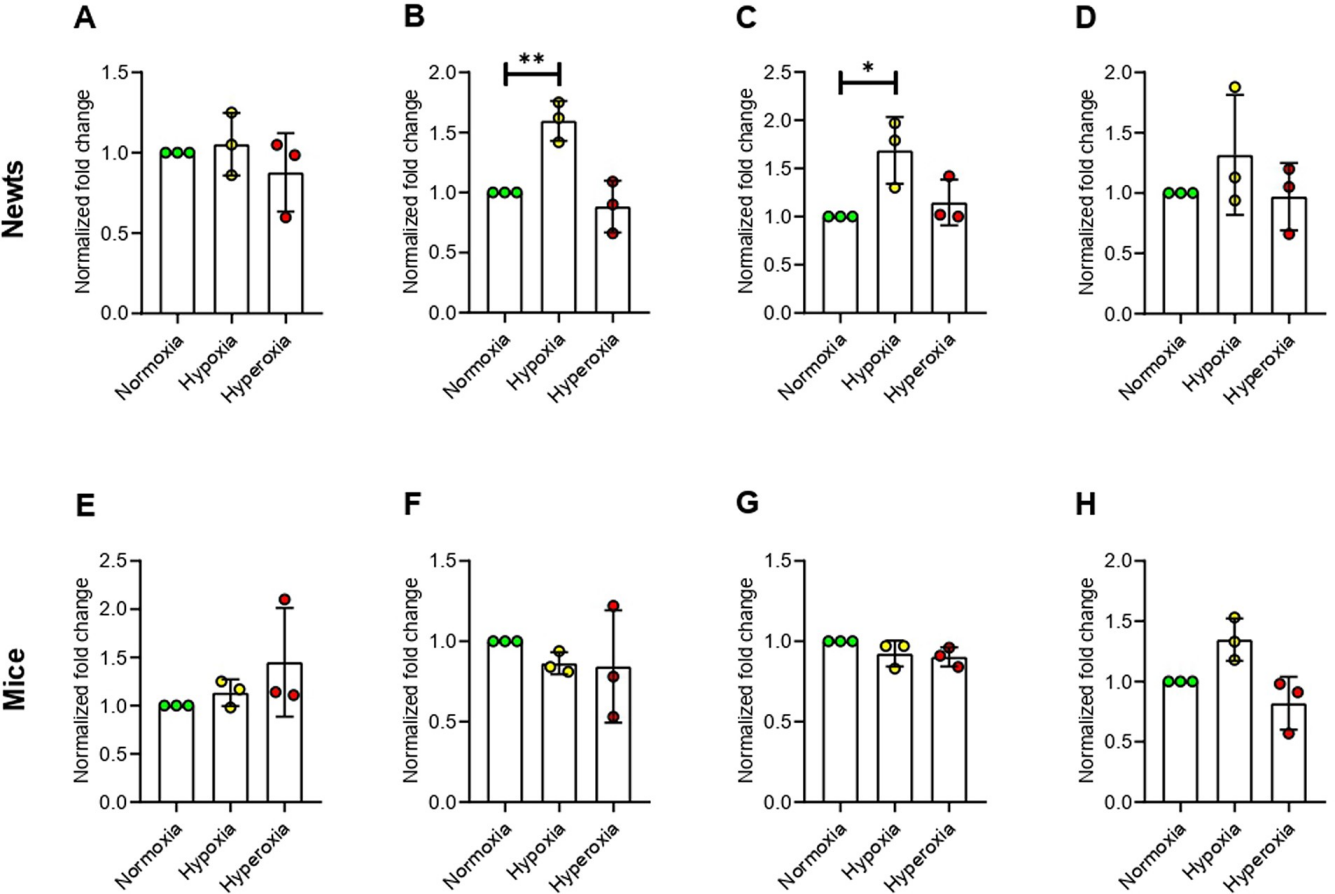
Effect of hypoxia and hyperoxia on genes that encode methylated CpG binding domain proteins (MBDs) in newts (A, B, C, and D) and mice (E, F, G, and H). A and E = methyl-CpG binding domain protein 1 (*Mbd1*), B and F = methyl-CpG binding domain protein 2 (*Mbd2*), C and G = methyl-CpG binding domain protein 3 (*Mbd3*), D and H = methyl-CpG binding protein 2 (*Mecp2*). Data are presented as mean ± SD. Experiments were done in triplicate. *p<0.05, **p<0.01.

No significant change was observed in methyl-CpG binding domain 1 (*Mbd1*) expression both in newts and mice exposed to hypoxia or hyperoxia (Fig 3A and 3E). Expression of methyl-CpG binding domain protein 2 (*Mbd2*) was increased significantly (*p*<0.01) by 1.60±0.17 fold in newts exposed to hypoxia (Fig 3B). However, hyperoxia did not affect expression of *Mbd2* in newts (Fig 3B). On the other hand, *Mbd2* expression was not changed in mice exposed to hypoxia and hyperoxia (Fig 3F). Methyl-CpG binding domain protein 3 (*Mbd3*) mRNA expression was also elevated by 1.69±0.35 fold in newts only at hypoxia (*p*<0.05 *vs*. normoxia, Fig 3C), but was not changed in mice (Fig 3G). The expression of methyl-CpG binding protein 2 (*Mecp2*) mRNA was not changed in both newts and mice exposed to hypoxia or hyperoxia (Fig 3D and 3H).

### Hypoxia up-regulates histone deacetylases (HDACs) in newts, but not in mice

**Fig 4:**
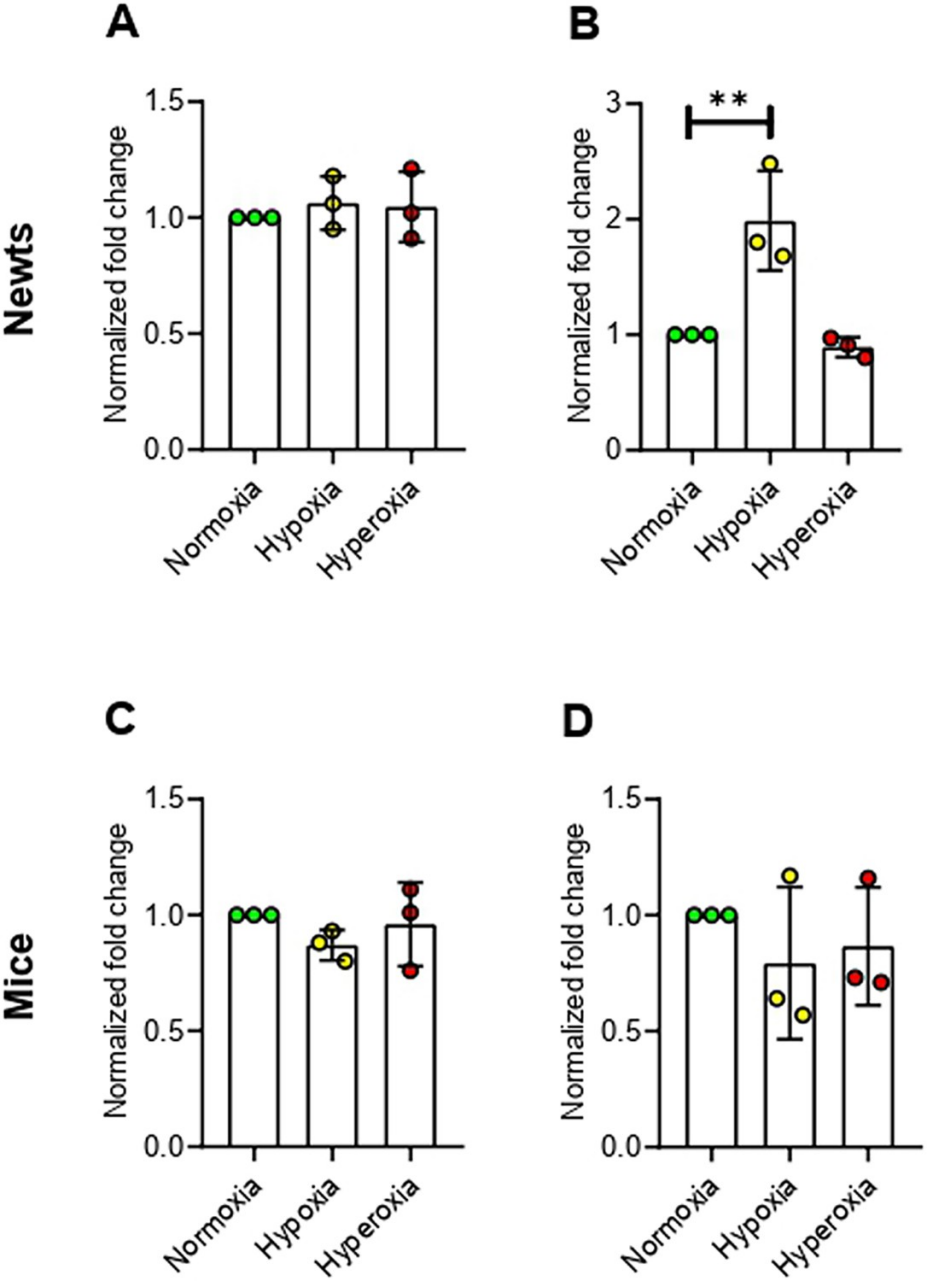
Effect of hypoxia and hyperoxia on genes that encode histone deacetylases (HDACs) in newts (A and B) and mice (C and D). A and C = histone deacetylase 1 (*Hdac1*), B and D = histone deacetylase 2 (*Hdac2*). Data are presented as mean ± SD. Experiments were done in triplicate. **p<0.01.

We did not find change in expression of histone deacetylase 1 (*Hdac1*) in either newts or mice exposed to hypoxia and hyperoxia (Fig 4A and 4C). However, histone deacetylase 2 (*Hdac2*) mRNA level was increased significantly (*p*<0.01) only in newts at hypoxia (Fig 4B and 4D).

### Hypoxia causes transcription inhibition in newts

**Fig 5:**
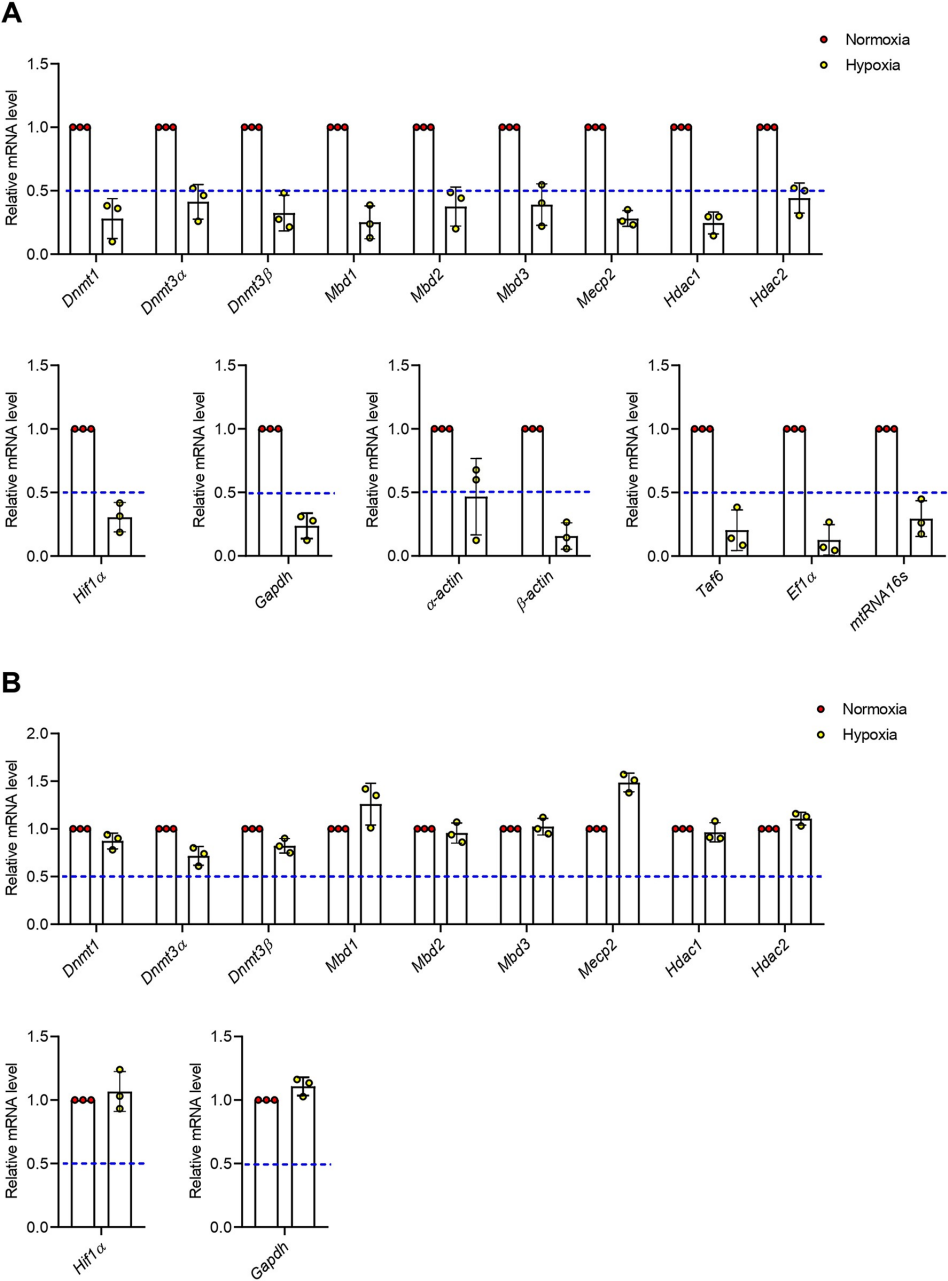
Relative (to control) mRNA level in lungs of newts (A) and mice (B) after hypoxia exposure. Data are presented as mean ± SD. Experiments were done in triplicate.

Our prior results regarding normalized fold change of genes including *Dnmt3α*, *Mbd2*, *Mbd3*, and *Hdac2* elevation suggest increased *de novo* methylation and subsequent transcription inhibition in newt lungs. Thus, we recalculated our data for evaluating relative (to control) expression of genes and found that all transcripts in newts were decreased dramatically but the extent of suppression is different (Fig 5A). In contrast, the relative gene expression in mice (Fig 5B) showed trends similar to normalized fold change.

## Discussion

Although we conducted a comparative study between newts and mice after exposing them to hypoxia and hyperoxia, we found differences in epigene expression patterns only under hypoxic condition. Hypoxia is detrimental to most animals and stimulates a complex tissue-specific microenvironment for oxygen homeostasis. Oxygen homeostasis under hypoxic condition is regulated by hypoxia-inducible factor 1 (HIF1) proteins [28]. There are two HIF1 subunits: HIF1α and HIF1β. Under normoxic and hyperoxic conditions, HIF1 proteins are degraded by prolyl hydroxylases (PHDs) [29]. Hypoxia protects HIF1 from degradation by PHDs. Thus, HIF1 proteins become stabilized and start its function as a transcription factor. This protein induces transcription of more than 60 genes which are involved in numerous pathways including but not limited to amino acid metabolism, angiogenesis, apoptosis, cell proliferation, cell survival, cytoskeletal structure remodeling, drug resistance, epithelial homeostasis, erythropoiesis, extracellular matrix metabolism, glucose metabolism, maintenance of vascular tone, motility of cells, nucleotide metabolism, and pH regulation [30]. Hypoxia not only stabilizes HIF1 protein but also induces expression of mRNA [31–33], which is later translated into protein to compensate for degraded protein for oxygen homeostasis. Here, we found an insignificant but slight elevation in mRNA level of *Hif1α* in newts exposed to hypoxia, but no change was observed in mice. Wiener et al. [31] showed that *Hif1α* mRNA synthesis was maximized in mouse at 1 hour and returned to basal level at 4 hours of exposure to 7% O_2_. In this study, we exposed mice to 8% O_2_ for 2 hours and did not find an elevation of *Hif1α* mRNA. We speculate that the mRNA level returned to basal state within 2 hours. This reversion of *Hif1α* mRNA to the basal level might be due to the stabilization of enough HIF1α proteins.

Generally, HIF1α, as a transcription factor, begins its role upon binding to the hypoxia response elements (HREs) on DNA. The core HRE sequence on DNA is CGTG, where methylation of cytosine occurs and initiates subsequent epigenetic regulation in response to hypoxia [34]. Upon binding to the HREs, HIF1α recruits histone acetyltransferases and promotes transcription of target genes [34]. Additionally, HIF1α proteins promote the upregulation of histone demethylase enzymes and cooperatively control gene expression [34].

However, the elevated expression of DNA methyltransferase 3 alpha (*Dnmt3α*) suggests increased *de novo* methylation at CpG sites on DNA in newts exposed to hypoxia [18,35]. Elevated DNA methylation is an indication of tolerance to oxygen starvation [18]. Methylated CpG sites at the promoter region directly hinder transcription factors from bind to the promoter region and impair transcription [18]. The methylated CpG sites attract nucleosome remodeling and histone deacetylation (NuRD) complex molecules, inducing subsequent transcription inhibition [18,36,37]. Interestingly, we found enhanced expression of NuRD complex protein-coding genes, including methyl-CpG binding domain protein 2 (*Mbd2*), *Mbd3*, and histone deacetylase 2 (*Hdac2*), in the lungs of newts exposed to hypoxia. MBD2 and MBD3 inhibit transcription indirectly by binding to methylated CpG sites at the promoter regions [18], while HDAC2 participates in removing acetyl groups from histones to form heterochromatin and negatively regulates transcription of certain genes [37]. Strikingly, enhanced *Hdac2* expression supports hypoxia tolerance [38] since it attenuates inflammation [39,40].

On the other hand, we found a reduction in *Dnmt1* and *Dnmt3α* mRNA in mice exposed to hypoxia. Reduced DNMTs expression resembles reduced methylation on DNA [41]. According to growing evidence, hypoxia decreases *Dnmt1* expression in mammals [41,42]. Loss of *Dnmt1* indicates a loss of control in maintaining the pre-existing physiological methylation pattern, leading to cell death at mitosis stage and genome instability [18,43]. It has been reported that deletion of *Dnmt3α* is lethal [44]. Collectively, the decreased expression of *Dnmt1* and *Dnmt3α* due to hypoxia is detrimental for mice [18,35,43,44].

Of note, using *Gapdh* as an internal control might be questionable as *Gapdh* itself is known as a target of HIF1α. Previous studies have shown that *Gapdh* expression in alveolar epithelial cells remains stable even after three hours at 0% O_2_ [45,46]. It has also been reported that *Gapdh* is not involved in hypoxia habituation in lung fibroblasts or smooth muscle cells [46]. In this study, hypoxia (8% O_2_) treatment for two hours did not affect *Gapdh* expression in mice lung (S1 Table), which is consistent with prior reports [45,46]. However, we observed unstable *Gapdh* expression (larger Cq values) in newts after exposure to hypoxia (S1 Table), which seems unusual compared to observations in mammals. The Cq values for the target epigenes were also unstable but parallel to the Cq values of *Gapdh*. Thus, we used *Gapdh* as an internal control because the data normalized by *Gapdh* showed the smallest individual variation. We also checked several other housekeeping genes, such as *α-actin*, *β-actin*, elongation factor 1 alpha (*Ef1α*), mitochondrial RNA 16S (*mtRNA16s*), and TATA-box binding protein associated factor 6 (*Taf6*), which are well-used as internal controls; however, all of them resulted in expression trends similar to *Gapdh*. Additionally, the Cq values for the later-mentioned housekeeping genes were also unstable but had parallelism to Cq value of *Gapdh* of the corresponding sample (S1 Table) and yielded higher individual variation among the data points.

To our surprise, the consecutive enhanced expression of *Dnmt3α*, *Mbd2*, *Mbd3*, and *Hdac2* suggests global transcription inhibition in lungs of newts [18]. Wijenayake and Storey [18] reported DNA hypermethylation and subsequent transcription inhibition in a tissue-specific manner in the semiaquatic turtle *T*. *scripta* elegans after exposure to anoxia. Due to anoxia exposure, enhanced DNMT1, DNMT2, MBD1, and MBD2 in liver and DNMT3α, DNMT3β, and MBD1 in white muscle of *T*. *scripta* elegans were observed [18]. Recently, global hypermethylation and subsequent transcription inhibition in brain and heart of goldfish due to chronic hypoxia exposure have also been reported [47]. Therefore, to prove the hypothesis of global transcription inhibition in lungs of our newt model, we performed relative (to control) gene expression analysis and found a dramatic decrease in all transcripts in newts. Transcription is one of the energetically expensive processes and requires a certain amount adenosine triphosphate (ATP). Inhibited *mtRNA16s* indicates suppression of oxidative phosphorylation (OXPHOS) [48]. As a consequence, a metabolic switch from OXPHOS to glycolysis is expected for energy homeostasis. Interestingly, we found a dramatic decrease in *Gapdh* mRNA, a glycolytic gene, instead of its elevation. Therefore, the decreased expression of *mtRNA16s* and *Gapdh* suggests metabolic suppression in newts. Additionally, the suppressed expression of a transcription factor *Taf6* is an indication of transcription inhibition. Such transcriptional inhibition in newt lungs indicates metabolic suppression, suggesting an adaptive mechanism for efficient hypoxia survival [13,18,47].

The limitation of this study lies in the fact that we did not optimize the ideal hypoxic condition for *P*. *waltl* by subjecting them to varying oxygen level, as mice from the same cohort used in this experiment died when exposed to less than 8% oxygen (data not shown). Strikingly, Sheafore et al. [49] studied the effects of hypoxia on *Desmognathus fuscus*, a lungless salamander species. They observed increased buccal activity in *D*. *fuscus* exposed to oxygen levels ranging from 5 to 8%, while there was no change in groups exposed to 10% or even 2% oxygen supply [49]. Though heart rate significantly increased at 10, 8, 6.5, and 5% oxygen exposure conditions, 2% oxygen exposure did not affect heart rate [49]. In addition to the heart rate, the apnea period was not impaired at 2% oxygen exposure, while apnea period was shortened in all other conditions (10, 8, 6.5, and 5% oxygen) [49]. Based on the findings by Sheafor et al. [49], optimizing an ideal hypoxia condition for newt might be challenging. Therefore, we chose to create a hypoxic environment at 8% oxygen based on findings by Sheafor et al. [49] and considering the hypoxia tolerance threshold (8% oxygen) observed in the mice from the same cohort.

In conclusion, we found opposite epigene expression patterns in lungs of newts and mice after hypoxia exposure. In the case of newts, the transcription inhibition or metabolic suppression in response to hypoxia might be another excellence of salamanders in addition to regeneration [24,50] and resistance to long-term starvation stress [51], senescence [52], and carcinogens [20,21] which are not well developed in adult mice. To make this differential epigene expression pattern clinically translational, further study to elucidate the specific mechanism of action involved in hypoxia tolerance of *P*. *waltl* is a prerequisite.

## Supporting information

S1 Table Cq values acquired from RT-qPCR. (XLSX)
